# The Protective Role of Vigor in Volunteering Against Somatic and Psychological Symptoms in EMDR Therapists

**DOI:** 10.11621/pir.2026.0106

**Published:** 2026-03-15

**Authors:** Alena A. Zolotareva, Julia S. Vitko, Elena V. Kazennaya

**Affiliations:** a HSE University, Moscow, Russia

**Keywords:** anxiety, applied psychology, depression, psychotherapy, somatic symptoms

## Abstract

**Background:**

Empirical evidence highlights that psychotherapists and applied psychologists experience stress, anxiety, depression, emotional exhaustion, secondary traumatic stress, and other symptoms of somatic and psychological distress. Although studies suggest that volunteering can protect somatic and psychological well-being in the general population, these relationships have not been investigated in mental health professionals.

**Objective:**

This study aimed to examine the role of volunteering and work-related characteristics in symptoms of somatic and psychological distress among psychotherapists and applied psychologists.

**Design:**

The study had a cross-sectional design. The participants were psychotherapists and applied psychologists who provided volunteer Eye Movement Desensitization and Reprocessing (EMDR) therapy sessions. One hundred and twenty volunteer EMDR therapists filled out the forms measuring somatic, anxiety, and depressive symptoms, job satisfaction, meaningfulness of work, and engagement in volunteering.

**Results:**

Correlation analyses showed that symptoms of somatic and psychological distress were negatively associated with job satisfaction, vigor in volunteering, and dedication in volunteering. Regression analyses revealed that vigor in volunteering was the only significant negative predictor of somatic (β = –.247, p = .049), anxiety (β = –.263, p = .004), and depressive symptoms (β = –.263, p = .034).

**Conclusion:**

This study contributes to our knowledge of the benefits of volunteering for the somatic and psychological well-being of EMDR therapists. From a practical standpoint, these findings suggest that psychotherapists and applied psychologists might be encouraged to frame pro bono work as a form of emotional and professional self-regulation. Furthermore, training programs could integrate pro bono activities into their curricula.

## Introduction

The mental and physical health of psychotherapists and applied psychologists is attracting increasing attention from researchers and practitioners. Empirical evidence highlights the fragility of mental well-being among these professionals: studies report an incidence of 5% for insomnia, 20% for stress, 8% for anxiety and depressive symptoms (Schaffler et al., 2024), as well as 40% for emotional exhaustion, 22% for depersonalization, and 19% for low levels of personal accomplishment (O’Connor et al., 2018). Between 19% and 81% of mental health professionals report a history of personal trauma, and between 19% and 70% exhibit symptoms of secondary traumatic stress (Henderson et al., 2025). Many practitioners complained of somatic symptoms including headaches, back pain, and gastroenteritis (Raquepaw & Miller, 1989; Rupert & Kent, 2007). Collectively, these findings indicate that psychotherapists and applied psychologists experience a high somatic and psychological burden.

The abundance of somatic and psychological symptoms is a common reason for the intention to leave the psychotherapeutic practice and profession (Garcia et al., 2014; Rosenberg & Pace, 2006). Thus, one-third of practitioners reported a strong intention to leave working as a psychotherapist in the current or near future (Aminihajibashi et al., 2022). Psychological factors associated with practitioners’ intention to leave the profession include physical and psychological exhaustion stemming from personal life, work demands, and client interactions, as well as job dissatisfaction, disturbances in professional identity, and diminished work engagement (Roth et al., 2024). Notably, somatic and psychological distress appear to play a particularly salient role in intentions to leave the profession among younger and less experienced psychotherapists and applied psychologists, who typically have not yet developed the capacity to conserve their emotional energy (McCormack et al., 2018).

Thus, ensuring the well-being of psychotherapists and applied psychologists emerges as an ethical and professional imperative. Recent findings indicate that over half of practicing psychiatrists express regret regarding their career choice, stating they would not select psychiatry again if afforded the chance (Gu et al., 2023). In light of these concerns, researchers have focused on identifying protective factors that may mitigate somatic and psychological distress among mental health professionals and reduce their risk of professional attrition. This growing body of work suggests that higher levels of self-compassion (McCade et al., 2021), compassion satisfaction (Aminihajibashi et al., 2022), psychological well-being (Van Hoy & Rzeszutek, 2022), personal accomplishment (McCormack et al., 2018), psychological resources (Medisauskaite et al., 2023), positive affect, and life satisfaction (Samios, 2018) are associated with greater health benefits and a stronger intention to remain in the psychotherapeutic profession.

Filling a gap in the literature, this study explores volunteering as a potential protective factor for the somatic and psychological well-being of mental health professionals. There is extensive evidence that volunteering is associated with a higher sense of belonging and social connectedness (Luque-Suárez et al., 2021; Musick & Wilson, 2003; Russell et al., 2019); greater self-esteem, happiness, and life satisfaction (Borgonovi, 2008; Morrow-Howell et al., 2003; Thoits & Hewitt, 2001); growing social and human capital (Kragt & Holtrop, 2019); lower loneliness and depressive symptoms (Mayers et al., 2024); less suicidal behavior, premature mortality, and functional impairment (Greenfield & Marks, 2004; Konrath et al., 2012; Rosato et al., 2019). However, evidence for these relationships comes primarily from the general population, and the impact of volunteering on the somatic and psychological well-being of psychotherapists and applied psychologists remains poorly understood.

In view of the foregoing, this study was aimed at examining the role of volunteering and work-related characteristics in symptoms of somatic and psychological distress among psychotherapists and applied psychologists.

## Methods

### Participants

The study had a cross-sectional design. In March–April 2023, the Organizing Committee of the Eye Movement Desensitization and Reprocessing (EMDR) Russia Association sent letters to members asking them to fill out an online questionnaire. The assessment was anonymous. All participants provided informed consent prior to participation. The inclusion criteria were: 1) completed training in EMDR therapy; 2) conducted group or individual volunteer EMDR sessions. The exclusion criteria were: 1) incomplete EMDR therapy training; 2) no experience providing group or individual volunteer EMDR sessions.

An *a priori* power analysis was performed using G*Power 3.1.9.7 to justify the sample size (Kang, 2021). Assuming a medium effect size (*f*^2^= .15), an α level of .05, and a desired power of .95 for a multiple regression model with ten predictors, the analysis indicated a minimum required sample of 89 participants.

Our final sample of 120 participants exceeded this threshold, thereby providing adequate statistical power for the primary analyses. Among the participants were 111 (93%) women and 9 (7%) men aged 24 to 67 years with work experience in psychology ranging from 1 to 35 years and volunteering experience in EMDR therapy from 1 month to 6 years (see *Table 1*).

### Measures

The survey contained socio-demographic questions (sex, age, work experience, and volunteering experience) and the following instruments.

*The Somatic Symptom Scale-8 (SSS-8; Gierk et al., 2014).* The SSS-8 contains 8 items assessing somatic burden based on self-reporting of specific somatic symptoms (*e.g.*, “headaches”, “dizziness”, “trouble sleeping”). Each symptom was examined with the Likert scale from 0 (“not at all”) to 4 (“very much”). We used the Russian version of the SSS-8 (Zolotareva, 2022). The sum score was calculated for the SSS-8, with a possible range from 0 to 32. A cut-offscore ≥ 12 shows high somatization (Gierk et al., 2014). In this study, Cronbach’s alpha was .75.

*The Generalized Anxiety Disorder-7 (GAD-7; Spitzer et al., 2006).* The GAD-7 includes 7 items measuring the severity of anxiety symptomatology on self-reporting of specific anxiety symptoms (*e.g.*, “trouble relaxing”, “feeling afraid as if something awful might happen”). Each symptom was evaluated with the Likert scale from 0 (“not at all”) to 3 (“nearly every day”). We used the Russian version of the GAD-7 (Zolotareva, 2023a). The sum score was calculated for the GAD-7, with a possible range from 0 to 21. A cut-offscore ≥ 10 indicates high anxiety (Spitzer et al., 2006). In this study, Cronbach’s alpha was .81.

*The Patient Health Questionnaire-9 (PHQ-9; Kroenke et al., 2001).* The PHQ-9 contains 9 items assessing the severity of depressive symptomatology on self-reporting of specific depressive symptoms (*e.g.*, “feeling tired or having little energy”, “trouble falling asleep or sleeping too much”). Each symptom was examined with the Likert scale from 0 (“not at all”) to 3 (“nearly every day”). We used the Russian version of the PHQ-9 (Zolotareva, 2023b). The sum score was calculated for the PHQ-9, with a possible range from 0 to 27. A cut-offscore ≥ 10 shows high depression (Kroenke et al., 2001). In this study, Cronbach’s alpha was .73.

*The Short Index of Job Satisfaction (SIJS; Judge et al., 2000).* The SIJS includes 5 items measuring general job satisfaction (*e.g.*, “Most days I am enthusiastic about my work”). Each item was evaluated with the Likert scale from 1 (“strongly disagree”) to 5 (“strongly agree”). In this study, we translated the SIJS into Russian and examined its internal consistency (Cronbach’s alpha was .56). The sum score was calculated for the SIJS, with a possible range from 5 to 25.

*The Work and Meaning Inventory (WAMI; Steger et al., 2012).* The WAMI contains 10 items assessing positive meaning (4 items to assess the degree to which people find meaning and purpose in their work, *e.g.*, “I have found a meaningful career”), meaning-making through work (3 items to examine the attitude towards work as something broader and meaningful, *e.g.*, “My work helps me make sense of the world around me”), and greater good motivations (3 items to evaluate the awareness that one’s own work benefits society and others, *e.g.*, “The work I do serves a greater purpose”). Each item was examined with the Likert scale from 1 (“absolutely untrue”) to 5 (“absolutely true”). In this study, we translated the WAMI and examined its internal consistency (Cronbach’s alphas were .69, .66, and .58 for positive meaning, meaning-making through work, and greater good motivations, respectively).

*The Utrecht Work Engagement Scale (UWES-9; Schaufeli et al., 2006).* The UWES-9 includes 9 items measuring vigor (3 items to assess feeling full of energy at work), dedication (3 items to examine the involvement in the work), and absorption (3 items to evaluate the happy immersion in work). Each item was evaluated with the Likert scale from 0 (“never”) to 6 (“always/every day”). We used the Russian version of the UWES-9 (Lovakov et al., 2017). For the aims of this study, we modified the instruction and asked the respondents to examine their engagement in volunteering (*e.g.*, “My volunteering makes me feel strong and vigorous”, “My volunteering inspires me”, “I feel happy when I am volunteering intensely”). In this study, Cronbach’s alphas were .76, .79, and .72 for vigor, dedication, and absorption, respectively.

### Data analysis

There were four stages of data analysis. First, we assessed the normality of the distribution using the Shapiro-Wilk test (Kim, 2013). Most variables exhibited a non-normal distribution. Second, we examined Cronbach’s alphas for the study variables. Since all variables were measured by less than 10 items, Cronbach’s alphas > .5 were considered acceptable (Pallant, 2010). Third, we calculated medians for continuous variables and frequencies and percentages for categorical variables. Fourth, we used correlation analyses and multiple regression analyses to examine the relationships between study variables. The relationships were considered significant at p < .05. Statistical analyses were conducted using Jamovi 2.6.26 and JASP 0.19.3.0.

## Results

*Table 1* shows the descriptive statistics of the participants.

**Table 1:** Descriptive statistics of the participants

**Characteristics**	**M**	**SD**	**Me**	**Skew**	**Kurt**	**SW-test**
Age, years	41.6	8.16	40	.76	.87	.952 (p < .001)
Work experience, months	118	96.6	84	1.06	.30	.878 (p < .001)
Volunteering experience, months	11.5	12.8	8	2.18	5.48	.735 (p < .001)
Job satisfaction	22.2	1.96	22	.66	.38	.939 (p < .001)
Positive meaning of work	18.9	1.52	20	1.69	.44	.745 (p < .001)
Meaning-making through work	14	1.45	15	1.27	.59	.726 (p < .001)
Greater good work motivations	13.8	1.51	14	1.28	1.25	.784 (p < .001)
Vigor in volunteering	12.8	2.5	13	.42	.31	.692 (p = .002)
Dedication in volunteering	15.3	2.26	16	1.18	1.83	.897 (p < .001)
Absorption in volunteering	13.3	2.69	14	.29	.07	.968 (p = .006)
Somatic symptoms	7.06	4.91	6	.76	.16	.943 (p < .001)
Anxiety symptoms	3.16	2.83	3	2.33	10.2	.816 (p < .001)
Depressive symptoms	4.58	3.30	4	1.15	1.09	.897 (p < .001)

*Note. Sum scores were calculated for all variables. M = mean; SD = standard deviation; Me = median; Skew = skewness; Kurt = kurtosis; SW-test = Shapiro-Wilk test. The standard error of skewness is .22; the standard error of kurtosis is .44.*

Generally, 3% of respondents had anxiety, 9% had depression, and 19% had somatization. The prevalence of specific symptoms ranged from 15% (chest pain or shortness of breath) to 87% (feeling tired or having low energy) for somatic symptoms; from 13% (restless) to 72% (nervous, anxious, on edge) for anxiety symptoms; and from 6% (suicidal thoughts) to 82% (tired or little energy) for depressive symptoms.

Correlation analyses revealed the following patterns. Somatic symptoms were positively associated with age, meaning-making through work, and greater good work motivations, and negatively associated with work experience, volunteering experience, job satisfaction, as well as vigor, dedication, and absorption in volunteering. Anxiety symptoms were positively associated with meaning-making through work and negatively associated with age, work experience, volunteering experience, job satisfaction, and vigor, dedication, and absorption in volunteering. Depressive symptoms were positively associated with age and negatively associated with job satisfaction, positive meaning of work, and vigor, dedication, and absorption in volunteering. *Table 2* illustrates these patterns.

**Table 2:** Associations of somatic, anxiety, and depressive symptoms with volunteering, job satisfaction, and job meaningfulness among EMDR therapists

**Characteristics**	**Somatic symptoms**	**Anxiety symptoms**	**Depressive symptoms**
Age, years	.074 (p = .420)	–.081 (p = .379)	.064 (p = .490)
Work experience, months	–.055 (p = .552)	–.100 (p = .279)	–.018 (p = .842)
Volunteering experience, months	–.072 (p = .435)	–.095 (p = .303)	–.031 (p = .738)
Job satisfaction	–.286 (p = .002)	–.185 (p = .044)	–.329 (p < .001)
Positive meaning of work	.009 (p = .926)	.009 (p = .920)	–.078 (p = .396)
Meaning-making through work	.108 (p = .242)	.074 (p = .421)	.024 (p = .796)
Greater good work motivations	.043 (p = .637)	.031 (p = .739)	.017 (p = .852)
Vigor in volunteering	–.324 (p < .001)	–.263 (p = .004)	–.353 (p < .001)
Dedication in volunteering	–.244 (p = .007)	–.173 (p = .059)	–.263 (p = .004)
Absorption in volunteering	–.180 (p = .049)	–.067 (p = .467)	–.145 (p = .115)

Given the exploratory nature of the study and the limited sample size, only predictors showing a statistically significant bivariate correlation with the dependent variable and demonstrating at least a small effect size (|r| ≥ .20) were entered into the regression model to avoid overfitting and ensure model parsimony.

*Model 1* examined job satisfaction, vigor in volunteering, and dedication in volunteering as predictors of somatic symptoms. Tolerance values were above .40 and VIF values were below 2.50 for all predictors, indicating no evidence of multicollinearity. The regression model was statistically significant (F^[3, 116]^ = 5.282, p = .002; R^2^ = .120, adjusted R^2^ = .097). Vigor in volunteering had a significant effect on somatic symptoms.

*Model 2* assessed vigor in volunteering as a predictor of anxiety symptoms. As only a single predictor was included in the model, multicollinearity was not a concern. The regression model was statistically significant (F^[1, 118]^ = 8.791, p = .004; R^2^ = .069, adjusted R^2^ = .061). Vigor in volunteering was negatively associated with somatic symptoms.

*Model 3* examined job satisfaction, vigor in volunteering, and dedication in volunteering as predictors of depressive symptoms. Tolerance values were above .40 and VIF values were below 2.50 for all predictors, indicating no evidence of multicollinearity. The regression model was statistically significant (F^[3, 116]^ = 6.777, p < .001; R^2^ = .149, adjusted R^2^ = .127). Vigor in volunteering had a significant effect on depressive symptoms.

*Table 3* illustrates these relationships.

**Table 3:** Predictors of somatic, anxiety and depressive symptoms

**Model**	**B**	**SE**	**95% CI**	**t-value**	**p-value**	**tolerance**	**VIF**
	*Model 1: Predictors of somatic symptoms*						
Job satisfaction	–.155	.282	(–.945; .171)	–1.373	.172	.598	1.674
Vigor in volunteering	–.247	.244	(–.970; .002)	–1.988	.049	.491	2.039
Dedication in volunteering	.017	.276	(–.510; .583)	.132	.895	.472	2.120
	*Model 2: Predictors of anxiety symptoms*						
Vigor in volunteering	–.263	.101	(–.498; .099)	–2.965	.004	n/a	n/a
	*Model 3: Predictors of depressive symptoms*						
Job satisfaction	–.200	.186	(–.706; .032)	–1.807	.073	.598	1.674
Vigor in volunteering	–.263	.162	(–.668; .027)	–2.150	.034	.491	2.039
Dedication in volunteering	.036	.182	(–.309; .414)	.287	.774	.472	2.120

*Note. β = beta standardized; n/a = not applicable.*

## Discussion

This study aimed to examine the role of volunteering and work-related characteristics in somatic and psychological distress among psychotherapists and applied psychologists.

We found that 3% of EMDR therapists had anxiety symptoms, 9% reported depressive symptoms, and 19% complained of somatic symptoms. These values were much lower than 25-43% for anxiety symptoms and 25-45% for depressive symptoms but were within a wide range of 8-68% for somatic symptoms in health care workers during the COVID-19 pandemic (Chen et al., 2022; Sahebi et al., 2021; Theocharis et al., 2023). Moreover, these values were lower than the 35% for anxiety symptoms and 27% for depressive symptom in medical students volunteering in the first months of the pandemic in Spain (Gómez-Durán et al., 2022). We believe that the low prevalence of mental health problems may be the result of not just volunteering, but of the high degree of mental health literacy and self-care among the medical students. In the general population, adequate mental health literacy decreased the risks of anxiety and depressive symptoms (Huang et al., 2021).

Greater vigor in volunteering was associated with fewer somatic, anxiety, and depressive symptoms. This relationship can be explained by at least three underlying mechanisms.

One potential mechanism involves the vigor in volunteering acting as a protective factor against burnout, which contributes to somatic and psychological distress. Recent research suggests that volunteering may be beneficial in preventing emotional burnout in healthcare workers and students, but may offer little benefit to those already suffering from it (Metzger et al., 2024). Burnout is strongly linked to anxiety and depression and can present with a range of somatic symptoms including fatigue, back pain, joint or limb pain, trouble sleeping, headaches, and alternating diarrhea or constipation (Gu et al., 2024; Hammarström et al., 2023). Therefore, volunteering may reduce somatic and psychological distress among psychotherapists and applied psychologists by preventing burnout. In contrast to routine clinical practice, volunteer counseling sessions are oriented toward collective restoration, fostering participants’ sense of belonging to a broader community (Sandberg et al., 2024).

Another possible mechanism is that volunteering helps psychotherapists and applied psychologists move beyond rigid role boundaries. By offering psychological support on a pro bono basis, they can renew their faith in the value of their profession and human connections, thereby developing and strengthening their professional identity. Similar processes underlie the formation of social and personality identities among volunteers (Camia et al., 2024; Gray & Stevenson, 2020; Wakefield et al., 2022). Rooted in values such as acceptance, autonomy and empowerment, honesty, humility, equality, respect, compassion, tolerance and openness to experience, the identities of mental health professionals are aligned with positive attitudes toward volunteering (Duggal & Sriram, 2021). Collectively, these findings suggest that vigor in volunteering may explain why a stable sense of identity is associated with less somatic and psychological distress (Raemen et al., 2023; Samaey et al., 2025).

Finally, another mechanism linking vigor in volunteering to somatic and psychological well-being may have a neurobiological basis. Evidence suggests that prosocial behavior activates distinct brain systems more robustly than reward-related stimuli (Wang et al., 2019). These systems, implicated in the processing of social information, include the insula, temporal lobe, and superior temporal gyrus. Social reward and positive social interactions contribute to the sense that a person’s life is meaningful (Kawamichi et al., 2016). Similar meaning-making processes may operate across other forms of prosocial engagement. For example, psychotherapists report deriving both self-oriented meaning, such as feeling gratified, fulfilled, and connected, and other-oriented meaning, such as helping others and contributing to a better world, from their work (Hill et al., 2017). This suggests that the experience of meaning may be a common psychological mechanism underlying the health benefits of diverse prosocial activities, including volunteering.

Taken together, the proposed mechanisms could help explain how volunteering is associated with somatic and psychological well-being among psychotherapists and applied psychologists, but they need to be examined in future research.

## Limitations

This study has several limitations. First, its cross-sectional design precludes causal inferences regarding the role of volunteering in the somatic and psychological wellbeing of psychotherapists and applied psychologists. Second, the data were based on self-reporting which may be subject to social desirability bias. Third, there is a risk of selection bias, as the mental health professionals who responded to the invitation may have had higher baseline levels of somatic and psychological well-being. Fourth, our sample consisted exclusively of EMDR therapists, which restricts the generalizability of the findings to other groups of psychotherapists and applied psychologists. Fifth, the sample was small and predominantly female, which may explain why many theoretically meaningful associations failed to reach statistical significance.

## Clinical implications

The finding that greater engagement in volunteering is associated with somatic and psychological well-being among mental health professionals has clear clinical implications. Psychotherapists and applied psychologists might be encouraged to frame pro bono work not merely as altruistic service but as a form of emotional and professional self-regulation that mitigates burnout and symptoms of somatic and psychological distress. Training programs could integrate pro bono activities into their curricula in order to simultaneously develop clinical competencies, strengthen professional identity, and enhance the sense of meaning in one’s professional work. Furthermore, clinics and professional associations may support such engagement, for example, by allocating a portion of clinicians’ working hours to providing free consultations to vulnerable populations.

## Conclusion

This study provides evidence that greater engagement in volunteering is associated with fewer somatic, anxiety, and depressive symptoms among psychotherapists and applied psychologists. However, given the cross-sectional nature of the data, causal inferences cannot be drawn. The relatively low prevalence of somatic and psychological distress in this sample may reflect the combined protective effects of professional expertise, mental health literacy, and prosocial engagement, yet a reverse causal pathway is also plausible: psychotherapists and applied psychologists in a more favorable psychological state may simply possess the personal resources and time to dedicate to volunteering. These findings underscore the potential role of volunteering not only as a form of social contribution but also as a component of a bidirectional relationship supporting mental health professionals’ own somatic and psychological well-being. The mechanisms underlying these relationships, including burnout prevention, professional identity reinforcement, and enhanced sense of meaning in one’s work, warrant further longitudinal investigation to clarify the directionality of these effects.
